# Preterm Delivery and Neonatal Deaths among Anaemic Pregnant Women in the Bolgatanga Metropolis of Ghana

**DOI:** 10.1155/2023/9865224

**Published:** 2023-06-15

**Authors:** Gideon K. Helegbe, Paul Aryee, Baba Sulemana Mohammed

**Affiliations:** ^1^Department of Biochemistry and Molecular Medicine, School of Medicine, University for Development Studies, Tamale, Ghana; ^2^Department of Nutritional Sciences, School of Allied Health Sciences, University for Development Studies, Tamale, Ghana; ^3^Department of Pharmacology, School of Pharmacy and Pharmaceutical Sciences, University for Development Studies, Tamale, Ghana

## Abstract

Preterm deliveries and neonatal deaths as functions of anaemia in pregnancy are of major public health interest. However, data on the prevalence of preterm deliveries and their association with mortality in anaemic pregnant women in the study area are scanty. Thus, the study sought to investigate the prevalence of preterm delivery and neonatal deaths among anaemic pregnant women in the Bolgatanga Regional Hospital in the Upper East Region of Ghana during the past five years. A retrospective study design was adopted, and data were gathered between March and May 2016. Records of women who were anaemic during any trimester of their pregnancy and delivered in the hospital within the last five years were included in the study. In all, two hundred (200) cases were reviewed. Data on the sociodemographic characteristics, health status, and birth outcome of participants were captured, and analyses were conducted using SPSS version 21 while considering significant differences at *p* < 0.05. The study revealed that more than half of the anaemic women (52.5%, *n* = 105) had preterm deliveries, while neonatal mortality was 8.5% (*n* = 17). The proportion of mothers who received dietary or medical intervention for the treatment of anaemia and the number of attendances to antenatal clinics were comparable between preterm and normal-term mothers (*p* > 0.05). Mothers with preterm deliveries had a higher risk of neonatal mortality (AOR = 13.66, 95% CI = 1.65–113.30, and *p*=0.015). This study has shown that anaemia in pregnancy increases the risk of preterm delivery and neonatal death. It is recommended that extra care be given to pregnant women with anaemia, while further studies are conducted with a larger sample size to substantiate the claims made in this study.

## 1. Background

Anaemia has remained a major public health concern in low and middle-income and high-income countries [1]. Globally, anaemia affects 1.62 billion people, which corresponds to 24.8% of the world's population [2], and vulnerable groups such as school-age children and pregnant women suffer the most from its consequences [2]. Among pregnant women, anaemia prevalence is reported to be very high globally, with about 40% being affected [3]. Poor birth outcomes such as stillbirth, preterm delivery, and low birth weight (LBW) are associated with anaemia and thus call for special attention [4]. In view of this, routine haemoglobin (Hb) assessments for pregnant women are performed during their visit for antenatal care (ANC) to monitor and manage anaemic conditions. As a routine, haematinics are given to these pregnant women, which have been shown to improve their anaemic situation [5].

Several factors are known to be associated with anaemia in pregnant women. These range from parasitic infections (helminths and malaria) [6], haemoglobinopathy [7], late initiation of ANC [8], poor diet and gestational age at first ANC [9], and low ANC visits (less than 4) [10] among others.

The World Bank report has indicated a gradual fall in the anaemia prevalence among Ghanaian pregnant women, from 59% in 1990 to 53% in 2016 [11]. According to regions, there were substantial disparities, ranging from a prevalence of 61% in the Greater Accra Region to 83% in the Northern Region [12]. However, data on the prevalence of preterm delivery and neonatal mortality among anaemic pregnant women in the study area are scanty. Thus, the aim of the study was to determine the prevalence of preterm deliveries and neonatal deaths among anaemic pregnant women in the Bolgatanga Regional Hospital in the Upper East Region of Ghana.

## 2. Methodology

### 2.1. Study Design and Study Area

**Figure 1:**
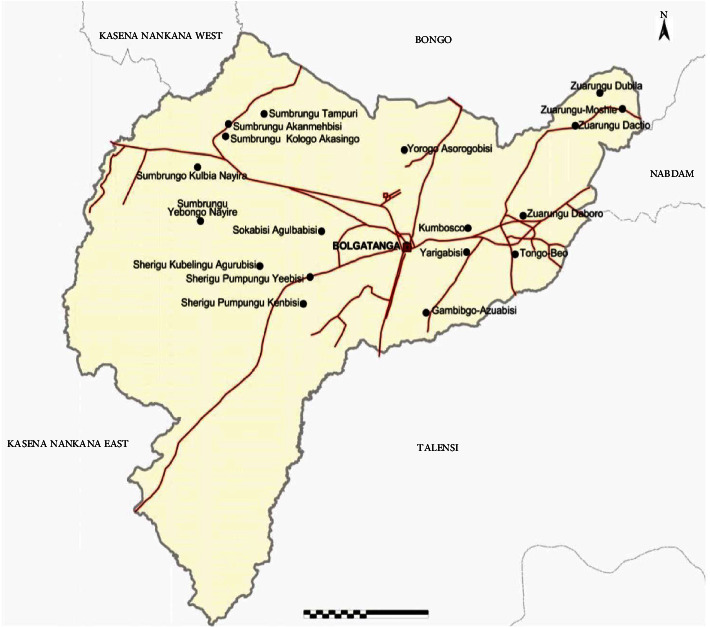
Map of Bolgatanga municipal [13].

A retrospective cross-sectional study design was carried out in March and April 2016 in the Bolgatanga Regional Hospital where most of the people in the region seek health care. Bolgatanga Regional Hospital is located in the Bolgatanga municipal of Ghana (Figure 1 as BRH) and serves the entire Upper East Region as well as some residents of the Upper West Region. Subjects were recruited based on the fact that they were anaemic during any period of their pregnancy. The gestational weeks of their children were considered to identify those who gave birth preterm (before the required 9 months) or normal term.

### 2.2. Sample Siz

With a prevalence of 53% of anaemia among Ghanaian pregnant women according to the World Bank Report [11], the Cochrane sample size formula [14] was used to estimate the minimum sample size for the study:

(1) $$\begin{array}{c} sample\;size\;\left(n_{o}\right)=\left(\frac{z2pq}{e2}\right)\text{,}\\ where\text{,}\\ z=1.96\text{,}\\ p=0.530\text{,}\\ q=1-0.530\\ =0.467\text{,}\\ e=0.05\text{,} \end{array}$$

where *z* = confidence interval; *e* = margin of error; *n*_*o*_ = sample size; and *p* = prevalence of anaemia of the study population at 53.0%, i.e., 0.530:

(2) $$\begin{array}{c} \left(n_{o}\right)=\frac{\left.1.96\;x1.96\;\left(0.530\right)\left(1-0.530\right)\right)}{0.05\;x\;0.05}\text{,}\\ \left(n_{o}\right)=\frac{3.8416\;\left(0.2475\right)}{0.0025}\text{,}\\ sample\;size\;\left(n_{o}\right)=380\text{.} \end{array}$$

Adding an attrition of 5% of the total sample size (380), which is 19 rounded to the nearest decimal, finally gave a total sample size of 399.

### 2.3. Study Population and Data Extraction

Anaemic pregnant women (Hb < 11 g/dL) comprised the study population. Data from the health records of these anaemic pregnant women and their delivery outcomes for the last five years (from 2011 to 2016) were extracted. The study subjects were further divided into preterm (those who delivered their babies before 37 weeks), and the rest were normal terms. In these two groups (preterm and normal term), the prevalence of mortality was determined. Maternal mortality was not considered in the recruitment criteria and thus was not included in the data for analysis.

### 2.4. Data Handling and Analysis

The data extracted were double entered to minimize data entry errors. Cleaning and coding were conducted before analysis. The data were analyzed using Microsoft Excel 2013 and Statistical Package for Social Sciences (SPSS) version 21 (IBM, Chicago Illinois, USA). The result was then presented in frequency, cross-tabulations, and diagrams as necessary, while *p* < 0.05 was set for statistical significance. Predictors of preterm delivery were evaluated by multinomial logistic regression analyses.

### 2.5. Ethical Consideration

To adhere to the highest ethical conduct of the study, serious consideration was made regarding study approval and participant consent to be part of the study. To this end, ethical clearance was sought from the joint School of Medicine and Health Sciences/School of Allied Health Sciences with SMSAHS/JIRB/0015 as the number. Permission was sought from the administrator and the head of the Gynaecology Unit of the BRH.

## 3. Results

### 3.1. Distribution of Sociodemographic Information among Mothers

A majority (55.5%, *n* = 111) of the mothers were aged between 21 and 30 years (Table 1). Majority (84.5.0%, *n* = 169) were employed, of which most (38.0%, *n* = 76) were artisans. Close to 64.0% (*n* = 128) were married, while the remaining unmarried mothers were either single, divorced, separated, or widows. A larger proportion (87.5%, *n* = 175) of the mothers had attained some level of formal education, of which the majority (68.5%, *n* = 137) had attained basic education (primary and junior high school (JHS)) (Table 1). It was realized that the recruited sample size was lower than the calculated sample size. This may be attributed to the inability to access data dating more than 5 years.

### 3.2. Clinical and Obstetric Characteristics of the Mothers

Anaemia was diagnosed by maternal haemoglobin levels of 11 g/dL [11]. The majority of mothers (73.6%, *n* = 148) had between 4 and 6 antenatal clinics (Table 2). Based on the clinical history and source of anaemia, dietary (76.5%, *n* = 153) or medical (23.5%, *n* = 47) interventions were prescribed for the mothers during their antenatal visit prior to delivery (Table 2). More than half of the mothers (52.0%, *n* = 104) had preterm delivery as they gave birth before 37 weeks of gestation (Table 2). Neonate mortality was observed in 8.0% (*n* = 16) of the mothers (Table 2).

### 3.3. Comparison of Socioeconomic, Clinical, and Obstetric Outcomes between Mothers with Preterm and Normal Delivery

No age differences were observed between preterm and normal-term mothers (*X*^2^ = 3.84 and *p*=0.281), Table 3. Similarly, no differences in occupational (*X*^2^ = 2.30 and *p*=0.129), marital (*X*^2^ = 0.39 and *p*=0556), and educational (*X*^2^ = 0.18 and *p*=0.669) statuses existed between preterm and normal-term mothers. The proportion of mothers who had received dietary or medical intervention for the treatment of anaemia was observed to be similar for both preterm and normal-term mothers (*X*^2^ = 0.73 and *p*=0.393). Moreover, the number of attendances to antenatal clinics did not differ between these mothers (*X*^2^ = 4.32 and *p*=0.157). However, the proportion of preterm women with neonatal mortality was higher than that of normal-term women (*X*^2^ = 12.16, *p* < 0.001), Table 3.

### 3.4. Factors That Predict Preterm Delivery among Mothers

The contributions of socioeconomic, clinical, and obstetric variables as predictors of preterm delivery were evaluated by multinomial logistic regression analyses. Before the analyses, multicollinearity issues among the predictor variables were resolved by a linear regression model, and predictors with a variance of inflation (VIF) of <2.000 were included in the multinomial regression model. In addition, Pearson correlation analyses were run to ensure there were no significant correlations among the predictor variables. The multinomial analyses showed that age, occupational, marital, and educational statuses did not influence the occurrence of preterm delivery (Table 4). Moreover, the number of antenatal clinics attended or the type of intervention used as a remedy for maternal anaemia did not influence the occurrence of preterm delivery (Table 4). However, mothers with preterm delivery had a higher risk of child mortality (AOR = 13.66, 95% CI = 1.65–113.30, and *p*=0.015, Table 4).

## 4. Discussion

Anaemia in pregnancy is of major public health concern, which calls for a pragmatic approach, but relevant data are lacking in the current study setting. The current study has shown that anaemia in pregnancy increases the risk of preterm delivery and neonatal death. Furthermore, preterm neonates are more likely to die within the first few days of birth.

Several factors have been associated with anaemia in pregnancy, including infectious diseases (malaria, helminths, hepatitis B, and HIV), low level of education, gestational age at the first ANC visit, and consumption of fish and snails [9, 15]. Meanwhile, preterm delivery and significant neonatal mortality were associated with anaemia in the current study; thus, anaemia in pregnancy has a great impact on birth outcomes.

The high risk of neonatal death in anaemic pregnant women may be attributed to multiple factors. One of them is the fact that anaemia in pregnancy results in low tolerance to loss of blood leading to impaired function and cardiac failure [16]. Related to this is iron deficiency, which increases oxidative damage to erythrocytes and the fetoplacental unit [17]. This deficiency in iron increases the risk of maternal infections. This further stimulates corticotropin-releasing hormone (CRH) production, making it a high-risk factor for preterm delivery on the basis that higher concentrations of CRH during labour also predict a shorter labour duration [18–20]. While high CRH is implicated in preterm delivery, it is important to state here that normal CRH concentration does not indicate that a normal delivery date is assured; this is because infections affecting the foetus and other problems can result in preterm delivery irrespective of the CRH level. Furthermore, there was a considerable difference in individual normal CRH concentrations. Nevertheless, the concentrations of CRH can significantly predict the duration of gestation. Meanwhile, it is observed that in humans, hypoxia, stress, preeclampsia, eclampsia, and inflammatory cytokines lead to increased placental CRH secretion [17].

With the relatively high preterm deliveries and neonatal deaths observed in the current study, it is important that interventions such as free distribution of insecticide-treated nets (ITNs) [21, 22], early initiation and improved antenatal care (ANC) [23], regular iron supplementation, screening of genetic diseases (e.g., G6PDd), and treatment of infectious diseases [24] be enhanced with focused and targeted education. The expectation is that, with such urgency and importance attached to these interventions, the level of anaemia in pregnancy will reduce significantly and consequently lower preterm delivery as well as neonatal deaths.

The current study has shown that the utilization of healthcare assessment was very beneficial to anaemic pregnant women. For example, women who attended the higher number of antenatal services recorded the least number of preterm births compared to those who did not attend antenatal frequently, while anaemic pregnant women who did not attend antenatal services frequently were more likely to give preterm babies. Furthermore, interventions that were initiated with anaemic pregnant women were also crucial in ensuring that the baby was born alive. Dietary and medical care given to these anaemic pregnant women improved the survival of the babies. It is at these ANC visits that pregnant women are educated and given targeted interventions after being screened. Thus, antenatal visits should be encouraged and promoted. Further studies are needed on a larger sample size to explore the extent to which parasitic infections [25], haemoglobinopathies [7], late initiation of ANC [8], and poor diet and gestational age [9] affect pregnancy outcomes.

The youthful age of the study population is encouraging, and the fact that most are employed and had some form of formal education as reported in another study [15] suggests that they are capable of managing their homes and can afford to get the necessary food items to improve their haemoglobin level. However, the majority of the study subjects (>60%), who are traders and artisans are noted to be busy and close late at night from their trading activities [26], are unable to attend their antenatal care services regularly and are not available at home for home health service delivery which is organized for pregnant women and young children on Thursdays and Fridays in the study area.

### 4.1. Limitations

A few limitations were observed in the current study. One of them is the fact that we were unable to get the required number for the study. This may influence the outcome of the findings and conclusions. Retrospective studies also have a deficiency of not getting certain clarifications one will want to get in the data captured, which the responses from the other study subject recruited at the time of the study could not address. Furthermore, anaemia may not be the only factor in determining neonatal death, although it may be strongly associated [27].

## 5. Conclusions and Recommendations

In conclusion, anaemia in pregnancy increases the risk of preterm delivery and neonatal death. Therefore, extra care should be given to pregnant women with anaemia, while further studies are conducted with a larger sample size to substantiate the claims made in this study.

## Figures and Tables

**Table 1:** Sociodemographic characteristics of the study participants (*n* = 200)^#^.

Category	Subcategory	*n* (%)	95% CI
**Age**	11–20	50 (25.0)	40, 64
	21–30	111 (55.5)	97, 125
	31–40	29 (14.5)	20, 40
	41–50	10 (5.0)	5, 17

**Occupational status**	Artisan	76 (38.0)	63, 90
	Trader	52 (26.0)	41, 65
	Farmer	26 (13.0)	18, 36
	Civil servant	9 (4.5)	4, 16
	Other^*∗*^	6 (3.0)	3, 12
	Unemployed^*∗∗*^	31 (15.5)	22, 42

**Marital status**	Married	128 (64.0)	114, 141
	Single	48 (24.0)	37, 61
	Divorced	8 (4.0)	4, 15
	Separated	1 (0.5)	0, 5
	Widow	15 (7.5)	9, 24

**Formal education**	None	25 (12.5)	17, 35
	Primary	43 (21.5)	32, 55
	J.H.S	94 (47.0)	80, 108
	S.H.S	22 (11.0)	14, 32
	Vocational/technical	8 (4.0)	4, 15
	Tertiary	8 (4.0)	4, 15

*n* (%): number and proportion; ^*∗*^hairdressers and seamstress; ^*∗∗*^housewife; ^#^recruited study subjects were lower than the estimated sample size. (95% CI): 95% confidence interval; S.H.S: senior high school.

**Table 2:** Clinical and obstetric information of the study participants (*n* = 200).

Category	Subcategory	*n* (%)	95% CI
Intervention when the mother was diagnosed anaemic	Dietary	153 (76.5)	141, 164
	Medical	47 (23.5)	36, 59

Number of antenatal visits	1–3	28 (14.0)	19, 39
	4–6	147 (73.5)	134, 158
	7–9	25 (12.5)	17, 35

Number of weeks the child was conceived before birth	Before 37 weeks	104 (52.0)	90, 118
	37 weeks and above	96 (48.0)	82, 110

Preterm or normal term	Normal term	96 (48.0)	82, 110
	Preterm	104 (52.0)	90, 118

Neonate mortality	Yes	16 (8.0)	10, 25
	No	184 (92.0)	175, 190

*n* (%): number and proportions; (95% CI): 95% confidence interval.

**Table 3:** Differences in socioeconomic and obstetric characteristics among mothers with preterm and normal-term delivery (*n* = 200).

Category	Subcategory	Normal delivery	Preterm delivery	(*X*^2^), ^*∗*^*p*
*Socioeconomic variables, n (%)*				
Age	11–20	22 (44.0)	28 (56.0)	3.84, 0.281
	21–30	58 (52.3)	53 (47.7)	
	31–40	10 (34.5)	19 (65.5)	
	41–50	6 (60.0)	4 (40.0)	

Occupational status	Unemployed	11 (35.5)	20 (64.5)	2.30, 0.129
	Employed	85 (50.3)	84 (49.7)	

Marital status	No	37 (51.4)	35 (48.6)	0.39, 0.556
	Yes	59 (46.1)	69 (53.9)	

Formal education	No	13 (52.0)	12 (48.0)	0.18, 0.669
	Yes	83 (47.4)	92 (52.6)	

*Clinical and obstetric outcomes, n (%)*				
Intervention for anaemia	Medical	20 (42.6)	27 (57.4)	0.73, 0.393
	Dietary	76 (49.7)	77 (50.3)	

Number of antenatal visits	1–3	9 (32.1)	19 (67.98)	4.32, 0.115
	4–6	72 (49.0)	75 (51.0)	
	7–9	15 (60.0)	10 (40.0)	

Neonate mortality	No	95 (51.6)	89 (48.4)	**12.16, <0.001**
	Yes	1 (6.3)	15 (93.8)	

*n* (%): number and proportions. ^*∗*^Analyzed using Pearson's chi-square test or Fisher's exact test. *χ*^2^ = Pearson's chi-square value. *p* significant at <0.05 (2-tailed). For formal education, yes: primary, JHS, SHS, vocational/technical, and tertiary, while no: none. For occupational status, employed: artisans, traders, farmers, civil servants, and others (hairdressers and seamstresses), while unemployed: housewife. For marital status, yes: married, while no: single, divorced, separated, and widow.The significance of the bold values is that the association is significant.

**Table 4:** Factors that predict preterm delivery among study participants.

Parameters		B	Standard error	Exp (B)/AOR	95% CI AOR	*p*
Age	11–20	−0.287	0.802	0.75	0.16, 3.62	0.721
	21–30	−0.236	0.736	0.79	0.19, 3.34	0.749
	31–40	0.666	0.793	1.95	0.41, 9.21	0.401
	41–50	0^b^	—	—	—	—

Occupational status	Unemployed	0.851	0.560	2.34	0.78, 7.02	0.129
	Employed	0^b^	—	—	—	—

Marital status	Not married	−0.449	0.360	0.64	0.32, 1.29	0.212
	Married	0^b^	—	—	—	—

Educational status	None	−0.329	0.488	0.72	0.28, 1.87	0.499
	Formal education	0^b^	—	—	—	—

Intervention for maternal anaemia	Medical	0.127	0.385	1.14	0.53, 2.42	0.742
	Dietary	0^b^	—	—	—	—

Number of antenatal visits	1–3	0.820	0.626	2.27	0.67, 7.75	0.190
	4–6	0.444	0.468	1.56	0.62, 3.89	0.343
	7–9	0^b^	—	—	—	—

Neonate mortality	Yes	2.614	1.079	13.66	1.65, 113.30	0.015

*p*: analyzed by multinomial logistic regression analyses and considered significant at < 0.05 (2-tailed). The reference category is normal-term delivery. b: parameter considered redundant and set to zero. B: regression coefficient. ExpB: exponentiation of B, which is the same as AOR: adjusted odds ratio. 95% CI: 95% confidence interval.

## Data Availability

Data can be made available upon reasonable request from the corresponding author.
